# Role of [18F] FDG PET-CT in detection of COVID-19 vaccine-associated hypermetabolic lymphadenopathy (VAHL) in lymphoma patients: with serologic testing correlation

**DOI:** 10.1186/s43055-022-00896-9

**Published:** 2023-02-02

**Authors:** Dena Abd El Aziz El Sammak, Rabab M. Abdelhay

**Affiliations:** 1 Egypt Radiodiagnosis Department, Zagazig University Hospital, Zagazig, Egypt; 2 Radiodiagnosis Department, Zagazig University Hospital, Egypt, Zagazig, Egypt

**Keywords:** PET-CT, COVID-19, VAHL, Lymphoma, Serology

## Abstract

**Background:**

COVID-19 vaccination of the population has a great importance, especially in oncological patients. The high incidence of vaccine-associated hypermetabolic lymphadenopathy (VAHL) makes a difficulty in the diagnosis of PET-CT of oncological patients. They should be vaccinated in the side opposite to the expected malignant LNs to avoid unnecessary biopsy and change in therapy. The aim of this study was to assess the role of PET-CT in detection of VAHL after the 2nd dose of Pfizer-BioNTech vaccine in lymphoma patients and compare the incidence of VAHL among lymphoma patients treated with B cell depletion therapy during the 6 months prior to vaccination and those treated > 6 months before vaccination.

**Results:**

This study comprised 120 lymphoma patients, referred for FDG PET/CT 1–3 weeks after the 2nd dose of Pfizer-BioNTech COVID-19 vaccine. Hypermetabolic LNs were identified in 55%. The incidence of VAHL in lymphoma patients treated with anti-CD20 antibody rituximab during the 6 months prior to vaccination (9%) was significantly lower compared with other lymphoma patients treated with anti-CD20 antibody rituximab > 6 months before vaccination (91%). The incidence and grades of VAHL are significantly high within the 1st week after the 2nd dose of Pfizer-BioNTech vaccine in patients younger than 60 years of age. Only 7 of 37 patients with negative serology had VAHL on PET-CT, whereas 10 of 26 patients with decreased anti-spike titers and 49 of 57 patients with increased anti-spike titers had VAHL on PET-CT.

**Conclusions:**

VAHL makes challenges in the interpretation of FDG PET/CT in oncology patients. Accurate data collection, regarding the time and site of COVID vaccination, is important to help radiologists in identifying the cause of abnormal nodal FDG uptake. We suggest to schedule FDG PET-CT for lymphoma patients at least 3 weeks after the 2nd dose of Pfizer-BioNTech vaccine.

## Background

COVID-19 pandemic has made a serious worldwide morbidity and mortality, to a large extent in immunocompromised and oncological patients, making mass vaccination of the population of great significance [1].

**Fig. 1:**
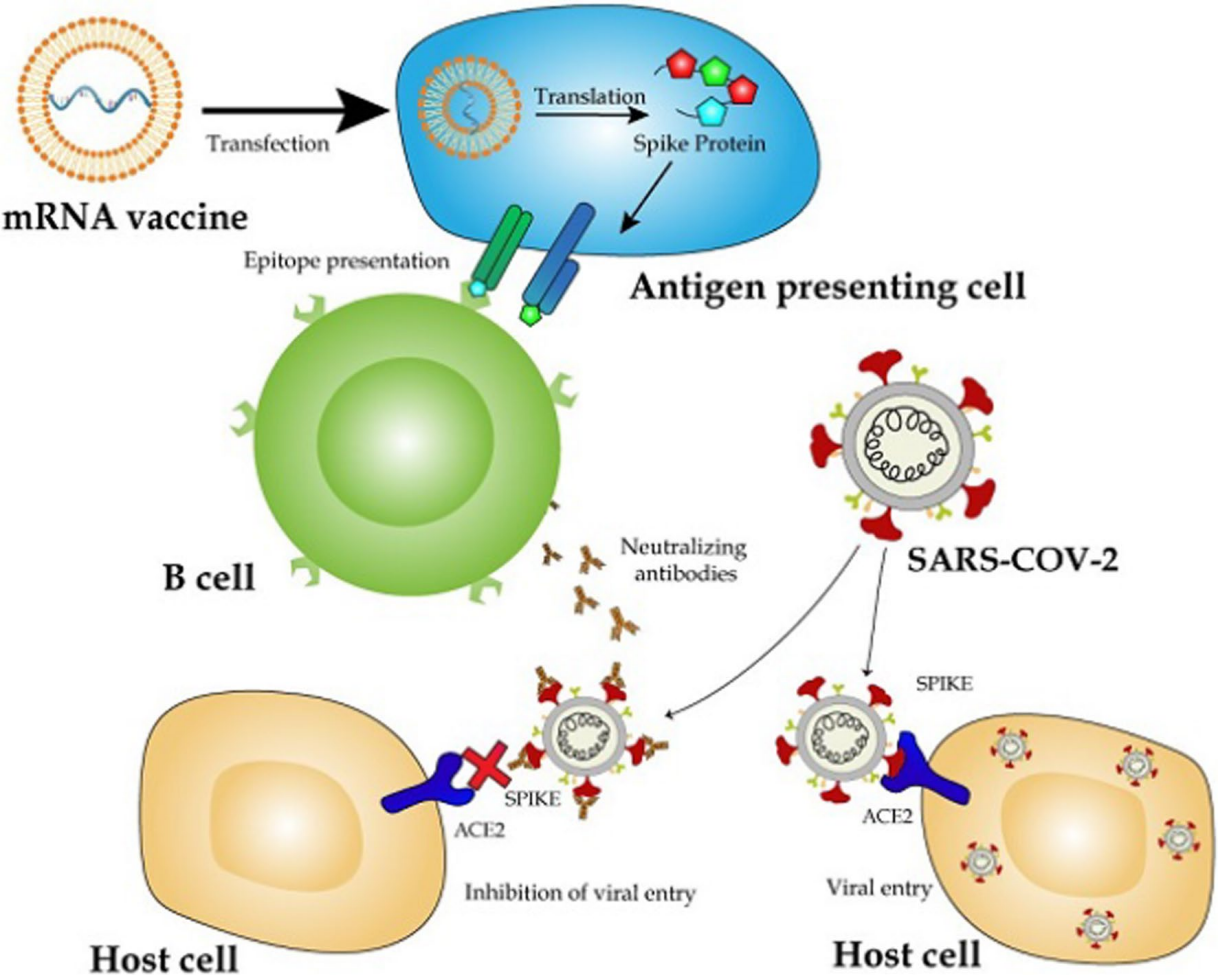
mRNA vaccines for COVID-19

Messenger ribonucleic acid (mRNA) vaccines are a novel technology that provides our bodies with a code to produce the virus spike protein. It stimulates a natural immune response, which depends on T cells and the production of neutralizing antibodies. Thus, in contrast to conventional vaccines, an mRNA vaccine does not contain any viral proteins itself, but only the information that our own cells need to produce a virus trait that triggers the desired immune response (Fig. 1) [2].

According to the previous experience with routine vaccinations, enlarged and sometimes painful axillary, supraclavicular and lower cervical lymph nodes have been described within 7 days following vaccination, affecting 4.8% of vaccine recipients [3].

[18F] FDG PET-CT has a significant role in detecting “hot” nodes, even if they are of normal size or located in nodal stations which may be missed on clinical examination [4].

The high incidence of VAHL makes a difficulty in the diagnosis of PET-CT of oncology patients, as the intensity measurement (SUVmax) of VAHL overlaps that of malignant lymphadenopathy, so the study was interpreted as equivocal [5].

Therefore, oncology patients should be vaccinated in the side opposite to the expected malignant LNs to avoid additional patient anxiety, unnecessary biopsy, excessive follow-up imaging studies and change in therapy [6].

In this study, we aimed to assess the role of FDG PET/CT in detection of VAHL after the 2nd dose of Pfizer-BioNTech vaccine in 120 lymphoma patients and compare the incidence of VAHL among lymphoma patients treated with B cell depletion therapy during the 6 months prior to vaccination and those treated > 6 months before vaccination.

## Methods

### Patients

This retrospective study was executed on 120 lymphoma patients (72 men and 48 women), referred from the clinical oncology department to the radiodiagnosis department in Zagazig university hospital during the period from October 2021 to April 2022. The lymphoma patients were referred for PET/CT imaging for: follow-up imaging in 54 patients, complaint of swelling, tenderness, or pain in the axilla in 61 patients, lower neck in 6 patients and supraclavicular region in 8 patients.

Inclusion criteria for this study were: lymphoma patients over 18 years old who performed FDG PET/CT 1–3 weeks after the 2nd dose of Pfizer-BioNTech COVID-19 vaccine.

Lymph nodes biopsy and histopathology were performed to the included 120 patients confirming benign reactive lymph nodes without any evidence of malignancy. Fifteen patients with malignant hypermetabolic lymphadenopathy (MHL) were excluded.

All patients were questioned about the date of the 2nd vaccine dose and the site of injection.

The included patients gave their written informed consent, and the protocol of this study was approved by the Committee of Ethics.

Among the 120 lymphoma patients (70 non-Hodgkin and 50 Hodgkin lymphoma), 36 patients (30%) were treated with anti-cd20 antibody rituximab during the 6 months prior to vaccination (median interval between the last therapy and vaccination was 2.41 months), while the other 84 patients were treated with anti-cd20 antibody rituximab > 6 months before vaccination (median interval 11.35 months).

A total of 120 patients underwent serologic testing following PET/CT imaging (IQR 15–21) days.

### 18 F-FDG PET/CT scanning (as shown in Figs. 2, 3, 4, 5, 6)

18 F-FDG PET/CT scans were performed in a private radiology center in Zagazig using an integrated PET/CT scanner (Ingenuity-TF 128; Philips, the Netherlands).

**Fig. 2:**
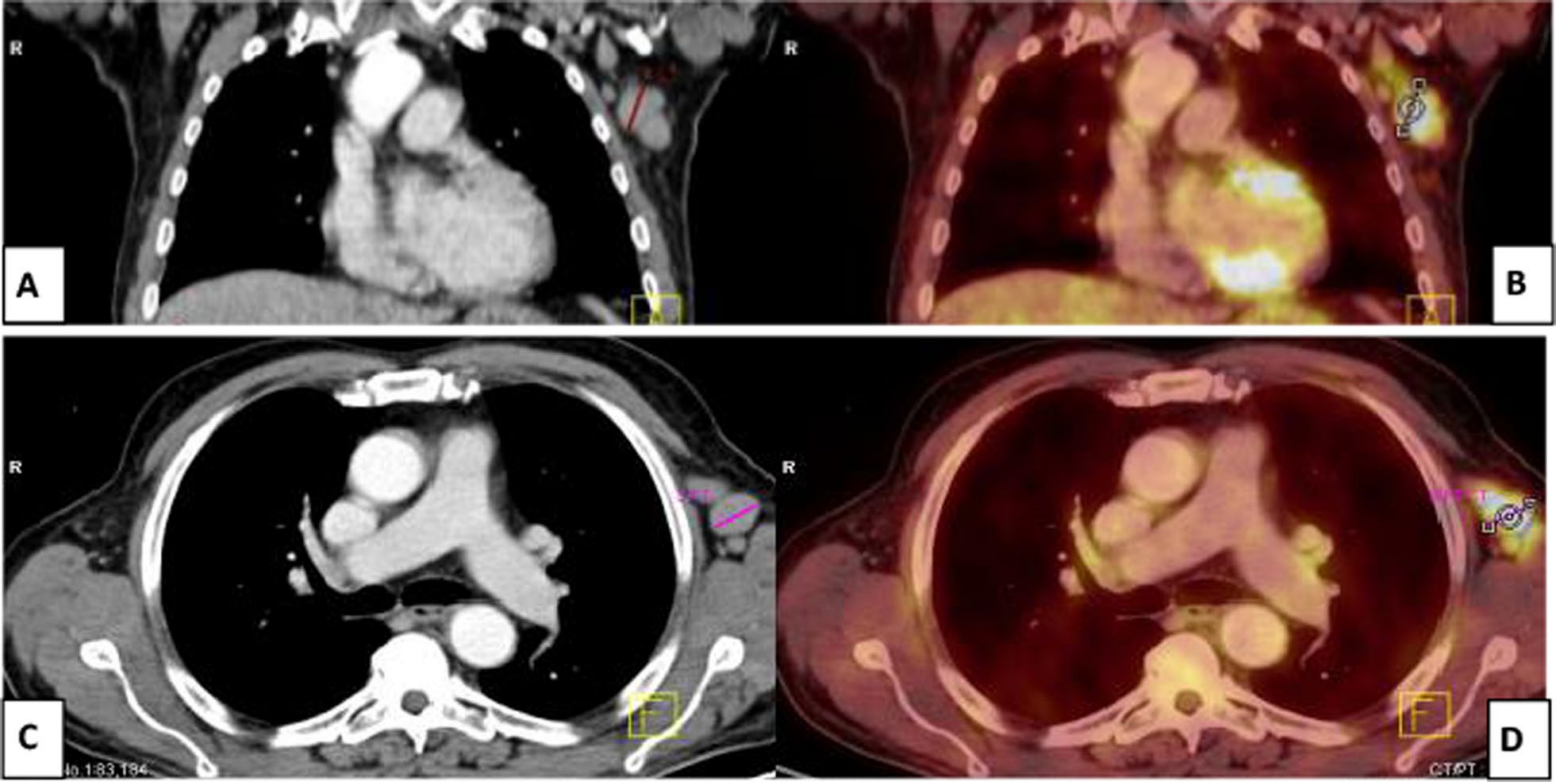
A 54-year-old male patient with Hodgkin’s lymphoma, treated with anti-cd20 antibody rituximab 4 months prior to vaccination. **A** Coronal and **C** axial CECT images revealed multiple enlarged left axillary LNs, largest measures 2.7 cm. **B** Coronal and **D** axial PET-CT images revealed enlarged and FDG-avid left axillary LNs of 3 SUVmax. At first, this was diagnosed as progression, ultrasound-guided biopsy-revealed reactive follicular hyperplasia with no malignancy. Clinical history revealed that he got the 2nd dose of Pfizer-BioNTech COVID-19 vaccine in his left arm 7 days before PET-CT study

**Fig. 3:**
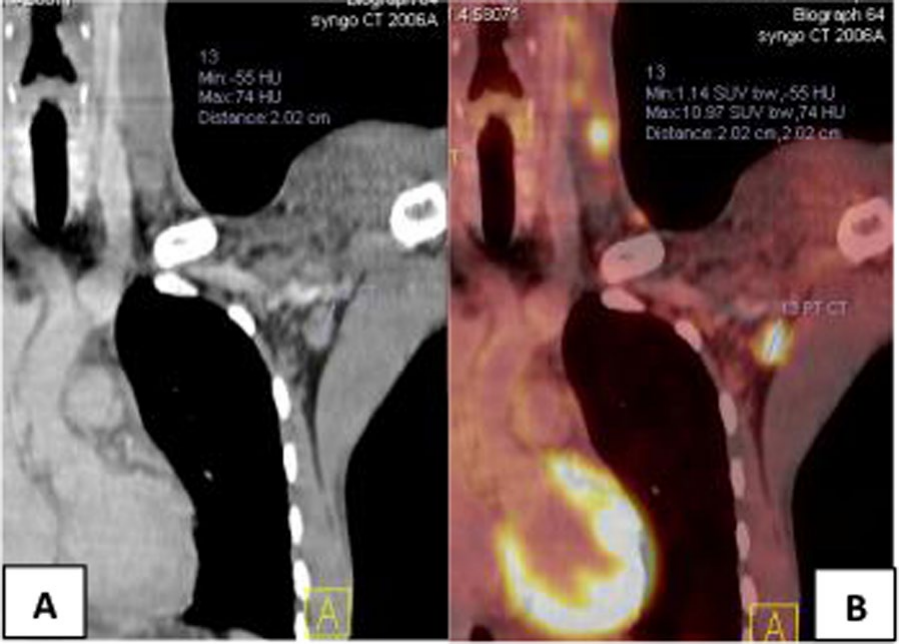
A 42-year-old male patient with NHL, treated with anti-cd20 antibody rituximab 10 months prior to vaccination. After 1 year of disease-free, PET/CT reported new left axillary, supraclavicular and lower cervical LNs. Clinical data revealed that he received the 2nd dose of Pfizer-BioNTech vaccine in his left arm 6 days prior to PET/CT. **A** Coronal CECT and **B** coronal PET/CT images revealed enlarged FDG-avid left axillary, supraclavicular and lower cervical LNs, largest measures 2 cm in left axilla of 11 SUVmax

**Fig. 4:**
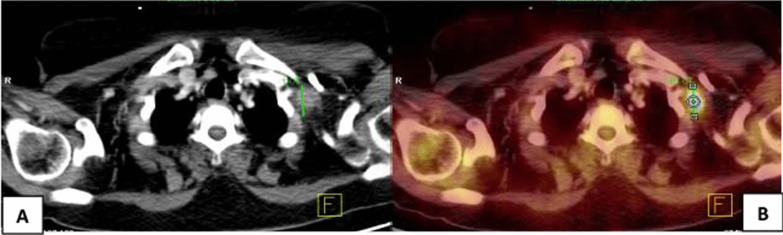
A 50-year-old female patient with NHL, treated with anti-cd20 antibody rituximab 5 months prior to vaccination. She was evaluated for left supraclavicular pain. Clinical data revealed that she had received the 2nd dose of Pfizer-BioNTech COVID-19 vaccine in her left arm 8 days prior to the PET/CT study. **A** Axial CECT image revealed enlarged left supraclavicular LN 2.6 cm. **B** Axial PET/CT image revealed enlarged FDG-avid left supraclavicular LN of 2 SUVmax

**Fig. 5:**
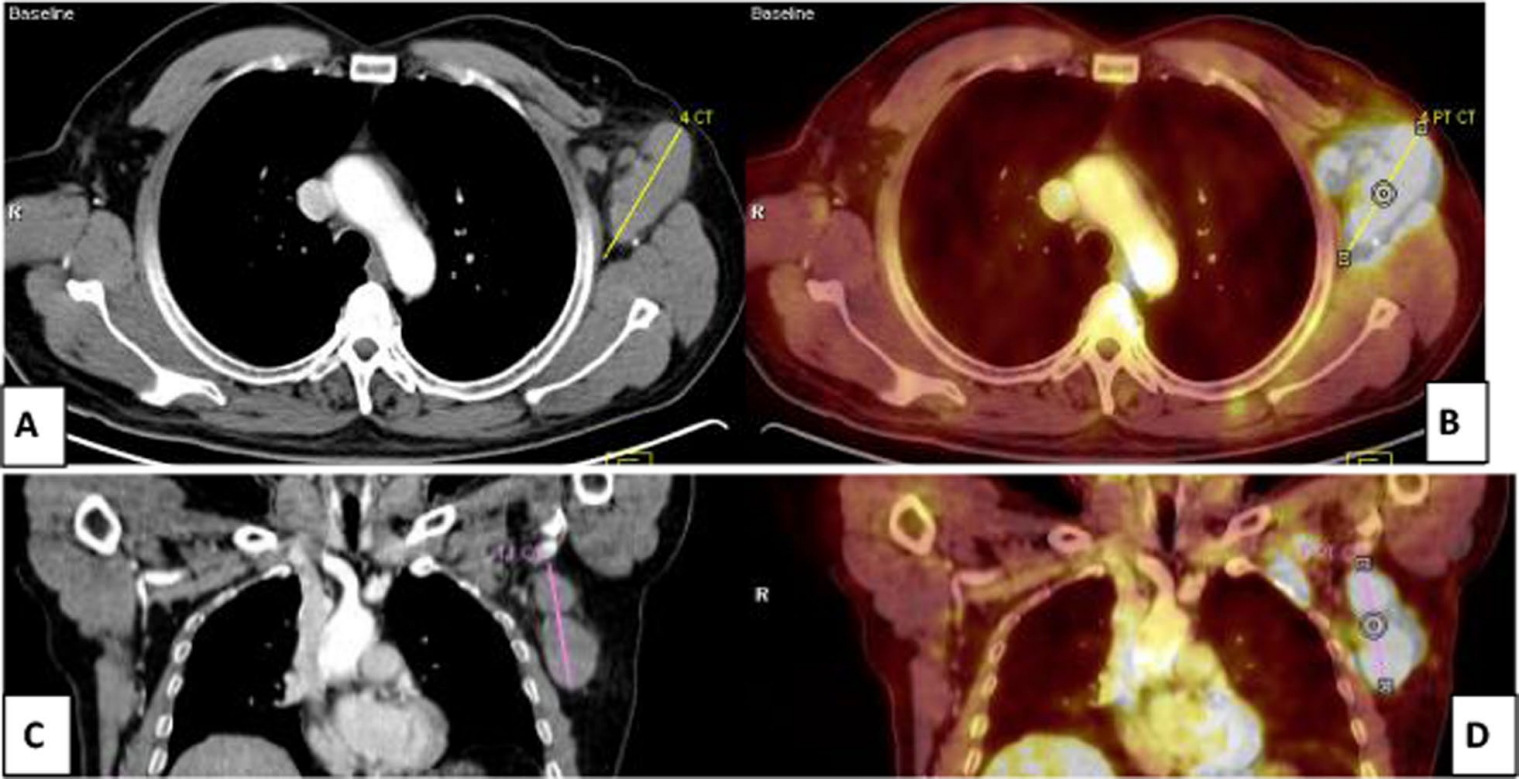
A 45-year-old male patient with NHL, treated with anti-cd20 antibody rituximab 25 months prior to vaccination. The most recent PET/CT showed enlarged amalgamated left axillary LNs. Further clinical data revealed that he got his 2nd dose of Pfizer-BioNTech vaccine 9 days before the PET-CT study. So, these findings were ascribed to recent vaccination. **A** Axial and **C** coronal CECT images revealed enlarged amalgamated left axillary LNs about 8 cm. **B** Axial and **D** coronal PET/CT images revealed FDG-avid left axillary LNs of 17 SUVmax

**Fig. 6:**
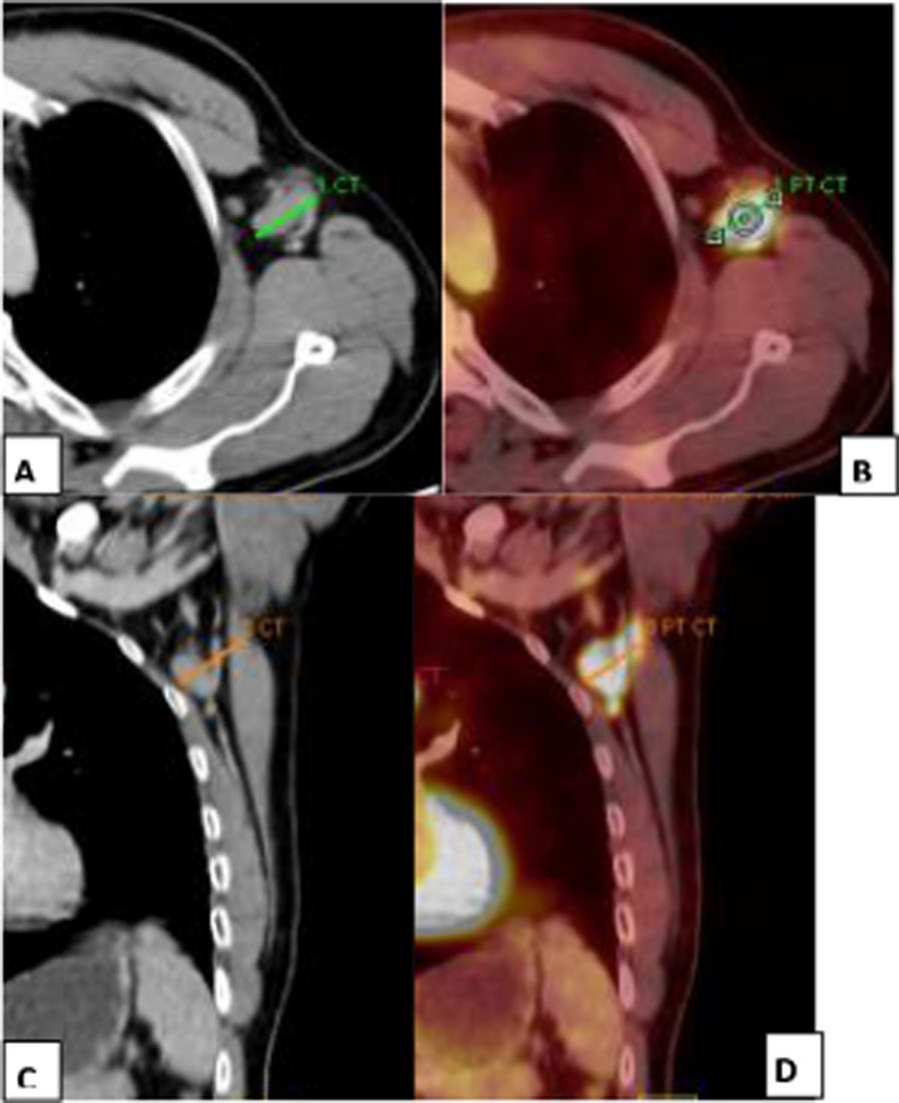
A 39-year-old male patient with NHL, treated with anti-cd20 antibody rituximab 3 months prior to vaccination. FDG PET/CT performed 7 days after the 2nd dose of Pfizer-BioNTech COVID-19 vaccine. **A** Axial and **C** coronal CECT images at the axillary level revealed enlarged left axillary LNs about 3.5 cm **B** Axial and **D** coronal PET/CT images revealed FDG-avid left axillary LNs with 3.7 SUVmax uptake

All patients should not consume any food, simple carbohydrates or liquids other than plain (unflavored) water for 4–6 h before examination. Adequate prehydration is important to ensure a sufficiently low concentration of FDG in the urine for radiation safety reasons. Coffee or caffeinated beverages are not recommended, because even if “sugarless” they may contain traces of simple carbohydrates and have the potential to induce excitant effects. Parenteral nutrition and intravenous fluids containing glucose should be discontinued 4 h before the FDG injection. Patients must avoid strenuous exercise 24 h before the PET/CT study.

Blood glucose level was measured 1 h before FDG injection as it should not exceed 150 mg/dl. All patients were asked to void before the examination.

The patient should be kept warm 60 min before FDG injection and continuing throughout the subsequent uptake period and examination to minimize FDG accumulation in brown fat (especially in winter or if the room is air-conditioned).

The examination was started with non-enhanced CT scan performed for attenuation correction; all patients were placed in a supine position with the arms resting above the head. They were scanned from the skull base down to the mid-thigh using the following parameters: a peak voltage of 120 kVp, 300 mAs, 12 mm table speed, 0.5 s rotation speed, and 0.75 mm collimation.

Following this, PET study was done using IV injection of 7–11 mCi of 18F-FDG (1 mCi/10 kg). During the injection of FDG, the patient should remain seated or recumbent and silent to minimize FDG uptake in muscles.

PET imaging started 45–60 min following tracer injection. 3D acquisition mode with 6–7 sequential table positions (3–5 min at each table position) was taken for entire patient scanning.

Then, enhanced CT scanning was done using low osmolar non-ionic contrast medium (Ultravist 370; Schering AG, Berlin-Wedding, Germany) containing 300 mg/ml of iodine with a volume range between 100 and 120 ml (1.5–2 ml/kg).

Using ordered-subset expectation software, attenuation correction was done using CT data. The attenuation-corrected PET images, CT images, and fused PET/CT images were interpreted at a workstation equipped with fusion software that provides multi-planar reformatted images for being reviewed in axial, coronal, and sagittal planes.

Semiquantitative analysis was performed by setting a spherical volume of interest (VOI) over the regions of interest and measuring the standardized uptake value (SUV) in the VOI.

### Image analysis

Images were classified as: positive VAHL if “hot” cervical or supraclavicular or axillary LNs ipsilateral to the vaccination site were detected, or negative VAHL if no “hot” nodes were detected.

Four grades of VAHL were recorded [6]:

*Grade 1*: mild FDG uptake intensity (SUVmax < 2.2).

*Grade 2*: moderate FDG uptake intensity (2.2 ≤ SUVmax < 4).

*Grade 3*: high FDG uptake intensity in normal-size nodes (SUVmax ≥ 4).

*Grade 4*: high FDG uptake intensity in enlarged nodes (SUVmax ≥ 4).

### Serology testing

A nucleocapsid protein IgG antibody test was done to ensure that all included patients had not been recently exposed to SARS-CoV-2.

Blood serum samples were analyzed using Elecsys® Anti-SARS-CoV-2S assay on the cobas e 601 (Roche Diagnostics) for the quantitative detection of IgG antibodies, aimed at the SARS-CoV-2 spike protein receptor-binding domain.

Antibody concentration of < 0.80 U/mL was considered as negative. Antibody concentration ≥ 0.80 U/mL but < 250 U/ mL was graded as low titer, and antibody concentration ≥ 250 U/mL was graded as high titer.

### Statistical analysis

All data were collected, tabulated, and statistically analyzed using SPSS 22.0 for windows (IBM Inc., Chicago, IL, USA). Continuous quantitative variables were expressed as the mean ± SD and median (range), and categorical qualitative variables were expressed as absolute frequencies (number) and relative frequencies (percentage). Continuous data were checked for normality by using Shapiro–Wilk test. Mann–Whitney U test was used to compare two groups of non-normally distributed data. Categorical data were compared using chi-square test or Fisher's exact test when appropriate. *P* value < 0.05 was considered statistically significant.

## Results

This study comprised 120 lymphoma patients, referred for PET-CT 1–3 weeks after the 2nd dose of Pfizer-BioNTech vaccine.

Thirty-six patients (30%) were treated with anti-cd20 antibody rituximab during the 6 months prior to vaccination (median interval between the last therapy and vaccination was 2.41 months); 83.3% were females and 16.7% were males, median age 66.50 years, scanned a mean of 11.91 ± 2.06 days after the 2nd vaccine dose.

The other 84 patients (70%) were treated with anti-cd20 antibody rituximab > 6 months before vaccination (median interval 11.35 months); 78.6% were males and 21.4% were females, median age 45.50 years, scanned a mean of 16.61 ± 3.61 days after the 2nd vaccine dose (Table 1).

**Table 1:** Comparison between patients treated ≤ 6 months before vaccination and those treated > 6 months before vaccination regarding basic characteristics

	Treated ≤ 6 months before vaccination (*N* = 36)		Treated > 6 months before vaccination (*N* = 84)	
Basic characteristics	No.	%	No.	%
*Sex*				
Male	6	16.7	66	78.6
Female	30	83.3	18	21.4
*Age (years)*				
Mean±SD	67.11 ± 4.86		47.77 ± 12.02	
Median (range)	66.50		45.50	
*Days between dose and PET-CT*				
Mean±SD	11.91 ± 2.06		16.61 ± 3.61	
**Location**				
*Axilla-level 1*				
Absent	31	86.1	28	33.3
Present	5	13.9	56	66.7
*Axilla-level 2*				
Absent	33	91.7	71	84.5
Present	3	8.3	13	15.5
*Axilla-level 3*				
Absent	35	97.2	75	89.3
Present	1	2.8	9	10.7
*Lower cervical*				
Absent	35	97.2	79	94
Present	1	2.8	5	6
*Supraclavicular*				
Absent	34	94.4	78	92.9
Present	2	5.6	6	7.1
*Days between PET-CT and serology*				
Mean±SD	18.63 ± 0.83		17.01 ± 1.54	
Median (range)	18 (18–21)		17 (15–21)	

Categorical variables were expressed as number (percentage); Continuous variables were expressed as mean ± SD and median (range)

Hypermetabolic LNs (median SUVmax 2.9 ± 1.3) were identified ipsilateral to the vaccine injection site in 66 of 120 vaccinated patients (55%).

The incidence of VAHL in lymphoma patients treated with anti-cd20 antibody rituximab during the 6 months prior to vaccination (9%) was significantly lower compared with other lymphoma patients treated with anti-cd20 antibody rituximab > 6 months before vaccination (91%).

The incidence and grades of VAHL were higher within the 1st week following the 2nd dose of Pfizer-BioNTech vaccine than the 2nd and 3rd weeks (Table 2).

**Table 2:** Comparison between patients treated ≤ 6 months before vaccination and those treated > 6 months before vaccination regarding HLN stratified by examination time

		Treated ≤ 6 months before vaccination		Treated > 6 months before vaccination			
Week	HLN	No.	%	No.	%	Test^a^	*p*-value
First week	No HLN	1	16.7	3	6.0	–	–
	Grade 1	3	50	20	40.0		
	Grade 2	2	33.3	19	38		
	Grade 3	0	0	7	14		
	Grade 4	0	0	1	2		
	Total	6	100	50	100		
Second week	No HLN	9	90	8	44.4	–	–
	Grade 1	1	10	6	33.3		
	Grade 2	0	0	2	11.1		
	Grade 3	0	0	1	5.6		
	Grade 4	0	0	1	5.6		
	Total	10	100	18	100		
Third week	No HLN	20	100	13	81.25	–	–
	Grade 1	0	0	2	12.50		
	Grade 2	0	0	1	6.25		
	Grade 3	0	0	0	0		
	Grade 4	0	0	0	0		
	Total	20	100	16	100		
Total	No HLN	30	83.3	24	28.6	111.071	< 0.001
	Grade 1	4	11.1	28	33.3		
	Grade 2	2	5.6	22	26.2		
	Grade 3	0	0	8	9.5		
	Grade 4	0	0	2	2.4		
	Total	36	100	84	100		

Categorical variables were expressed as number (percentage)

^a^Chi-square test; *p* < 0.05 is significant

Cases younger than 60 years showed higher incidence and grades of VAHL than those older than 60 years (Table 3).

**Table 3:** Comparison between patients treated ≤ 6 months before vaccination and those treated > 6 months before vaccination regarding HLN stratified by age group

		Treated ≤ 6 months before vaccination		Treated > 6 months before vaccination			
Age group	HLN	No.	%	No.	%	Test^a^	*p*-value
≤ 60 years	No HLN	9	60	6	10	–	–
	Grade 1	4	26.7	24	40		
	Grade 2	2	13.3	20	33.33		
	Grade 3	0	0	8	13.33		
	Grade 4	0	0	2	3.33		
	Total	15	100	60	100		
> 60 years	No HLN	21	100	18	75	34.605	< 0.001
	Grade 1	0	0	4	16.7		
	Grade 2	0	0	2	8.3		
	Grade 3	0	0	0	0		
	Grade 4	0	0	0	0		
	Total	21	100	24	100		
Total	No HLN	30	83.3	24	28.6	111.071	< 0.001
	Grade 1	4	11.1	28	33.3		
	Grade 2	2	5.6	22	26.2		
	Grade 3	0	0	8	9.5		
	Grade 4	0	0	2	2.4		
	Total	36	100	84	100		

Categorical variables were expressed as number (percentage)

^a^Chi-square test; *p* < 0.05 is significant

### The relationship between VAHL and post-vaccination antibody secretion

In 120 lymphoma patients, post-vaccination [18F] FDG PET-CT studies and serologic analysis were performed. The median interval between PET-CT and serologic analysis in patients treated during 6 months before vaccination and those treated > 6 months was 18 (IQR 18–21) days and 17 (IQR 15–21) days, respectively.

The serology results were negative in 37 patients (median antibodies concentration 0.30 U/mL), 26 patients had decreased anti-spike titers (median antibody concentration 10.8 U/mL), and 57 patients had increased anti-spike titers (median antibody concentration 952.4U/mL). Only 7 of 37 patients with negative serology had VAHL on PET-CT, whereas 10 of 26 patients with decreased anti-spike titers and 49 of 57 patients with increased anti-spike titers had VAHL on PET-CT (Table 4).

**Table 4:** Relationship between VAHL and serology

	VAHL					
	Absent (*N* = 54)		Present (*N* = 66)			
Serology	No.	%	No.	%	Test^a^	*p*-value
Negative	30	55.6	7	10.6	87.587	< 0.001
Low anti-spike titres	16	29.6	10	15.2		
High anti-spike titres	8	14.8	49	74.2		

Categorical variables were expressed as number (percentage)

^a^Chi-square test; *p* < 0.05 is significant

## Discussion

On September 30, 2021, Egypt received the 1st donation of Pfizer vaccines, gifted from the US Government and 8.25 million people in Egypt had received one or more doses of Pfizer vaccine [7].

Knowing mRNA vaccination-related secondary effects is essential for radiologists and oncologists to prevent false interpretations during diagnostic imaging procedures [8].

18F-FDG PET/CT is a nonspecific nuclear imaging tool, used mainly for the detection and staging of malignant tumors, the follow-up of oncologic patients, and the assessment of response to treatment of different tumors. However, FDG uptake can be seen in sites of active inflammation and infection [9, 10].

In this study, hypermetabolic LNs were identified ipsilateral to the vaccine injection site in 66 of 120 vaccinated patients (55%).

Cohen et al. [1] mentioned that the incidence of VAHL was 54% within 3 weeks after the 2nd dose of mRNA vaccine. However, 36.4% and 47.5% had VAHL on their FDG PET-CT studies following the first and third vaccine doses, respectively.

Addeo et al. [11] stated that in the Pfizer-BioNTech COVID-19 vaccine trial, the rate of VAHL was reported to be 0.3% among vaccine recipients versus < 0.1% among placebo group.

Treglia et al. [12] reported multiple PET/CT findings post-mRNA vaccination; increased 18F-FDG uptake in axillary, subpectoral, supraclavicular, lower cervical LNs and deltoid muscle ipsilateral to the vaccination site, also diffuse splenic FDG uptake was less frequently reported.

In some patients, reactive LNs may show high FDG uptake and normal size, because functional alterations may precede morphological abnormalities [13].

In this study, the incidence of VAHL in lymphoma patients treated with anti-cd20 antibody rituximab during the 6 months prior to vaccination (9%) was significantly lower compared with other lymphoma patients treated with anti-cd20 antibody rituximab > 6 months before vaccination (91%).

Cohen et al. [6] observed significant lower rates of VAHL in recently treated patients with anti-cd20 antibodies; this indicates that VAHL is a reflection of germinal center (GC) B cell proliferation as a part of an early humoral response to vaccines.

Also, Bingham et al. [14] mentioned that rheumatic patients treated with rituximab had low responses to pneumococcal vaccines. This result was similar to that mentioned by Eisenberg et al. [15] on Influenza vaccines.

Sonani et al. [16] reported that clinicians should recommend the timing of COVID-19 vaccines relative to anti-cd20 antibody rituximab schedule.

In this study, the incidence and grades of VAHL were higher within the 1st week following the 2nd dose of Pfizer-BioNTech vaccine than the 2nd and 3rd weeks.

Ahn et al. [17] stated that VAHL was identified in 82.5% of PET/CT scans performed within the first 5 days from vaccination.

Also, Baden et al. [18] reported that the incidence of VAHL in PET/CT performed 11–15 days from vaccination was 17.6% and dropped to 2.8% in those performed ≥ 16 days from vaccination.

Becker et al. [19] advised scheduling routine radiologic imaging before or at least 6 weeks after the final vaccination dose to reduce false positive results.

McIntosh et al. [4] suggested performing PET/CT at least 2 weeks after vaccination in patients with a cancer for which interpretation is anticipated to be potentially impacted by vaccination, but optimally 4–6 weeks after vaccination given increased immunogenicity of mRNA vaccines and potentially longer time for resolution than lymphadenopathy after other types of vaccines.

Ozütemiz et al. [20] stated that the incidences of all-grade VAHL and grade 3–4 VAHL were 47.5% and 8.9%, respectively. Grade 3–4 VAHL was observed on 28.1% of studies performed within the first 5 days from vaccination, compared with 0% of studies done ≥ 6 days from vaccination, *P* value < 0.01. This result was similar to that mentioned by Skawran et al. [21].

In this study, cases younger than 60 years showed higher incidence and grades of VAHL than those older than 60 years.

Baden et al. [18] stated that patients > 65 years old had weaker vaccine-induced immunity, as VAHL were detected in 8.4% after the 2nd dose.

In this study, the serology results were negative in 37 patients, 26 patients had decreased anti-spike titers, and 57 patients had increased anti-spike titers. Only 7 of 37 patients with negative serology had VAHL on PET-CT, whereas 10 of 26 patients with decreased anti-spike titers and 49 of 57 patients with increased anti-spike titers had VAHL on PET-CT.

After anti-SARS-CoV-2 mRNA vaccination, antigen-presenting cells (APCs) migrate to regional LNs and elicit cellular response forming cytotoxic T lymphocytes which destroy infected cells, and humoral response forming mature B cells (antibody-secreting plasma cells) and memory B cells [22].

Özütemiz et al. [20] mentioned in his study on 54 cases with hematologic malignancy having post-vaccination PET-CT and serologic testing that incidence of VAHL was the highest among cases with increased anti-spike titers. This positive correlation suggests that VAHL indicates an effective humoral response and a higher likelihood of antibodies production.

The thymus is a lymphoid organ that plays a cardinal role in the development of the immune system during childhood; it gradually involutes throughout maturation, yet maintains the ability to regrow [23]. Sabri et al. [23] suggest that thymic hyperplasia is an immune response to the viral infection. Samir et al. [24] stated that COVID-19 patients with poor thymic function need higher doses of COVID-19 vaccinations.

## Conclusions

VAHL makes challenges in the interpretation of FDG PET/CT, affecting disease staging and assessment of treatment response. Radiologists’ awareness of VAHL is important to prevent FDG PET/CT misinterpretation. Accurate data collection, regarding the time and site of COVID-19 vaccination, is important to help radiologists in identifying the cause of abnormal nodal FDG uptake. We suggest to schedule FDG PET-CT for lymphoma patients at least 3 weeks after the 2nd dose of Pfizer-BioNTech vaccine.

## Data Availability

The datasets used and analyzed during the current study are available from the corresponding author on reasonable request.

## Abbreviations

APCs: Antigen-presenting cells
COVID-19: Coronavirus disease 2019
dl: Deciliter
18F-FDG: 18F-fluorodeoxyglucose
GC: Germinal center
IgG: Immunoglobulin G
IQR: Interquartile range
IV: Intravenous
kg: Kilogram
kVp: Kilovoltage peak
LNs: Lymph nodes
mAs: Milliampere-seconds
mg: Milligram
MHL: Malignant hypermetabolic lymphadenopathy
ml: Milliliter
mm: Millimeter
mRNA: Messenger ribonucleic acid
PET/CT: Positron emission tomography/computed tomography
SUV: Standardized uptake value
US: United States
VAHL: Vaccine-associated hypermetabolic lymphadenopathy
VOI: Volume of interest
